# Genome-wide Transcriptional Profiling of Appressorium Development by the Rice Blast Fungus *Magnaporthe oryzae*

**DOI:** 10.1371/journal.ppat.1002514

**Published:** 2012-02-09

**Authors:** Darren M. Soanes, Apratim Chakrabarti, Konrad H. Paszkiewicz, Angus L. Dawe, Nicholas J. Talbot

**Affiliations:** 1 College of Life and Environmental Sciences, University of Exeter, Exeter, United Kingdom; 2 Department of Biology, New Mexico State University, Las Cruces, New Mexico, United States of America; University of Melbourne, Australia

## Abstract

The rice blast fungus *Magnaporthe oryzae* is one of the most significant pathogens affecting global food security. To cause rice blast disease the fungus elaborates a specialised infection structure called an appressorium. Here, we report genome wide transcriptional profile analysis of appressorium development using next generation sequencing (NGS). We performed both RNA-Seq and High-Throughput SuperSAGE analysis to compare the utility of these procedures for identifying differential gene expression in *M. oryzae*. We then analysed global patterns of gene expression during appressorium development. We show evidence for large-scale gene expression changes, highlighting the role of autophagy, lipid metabolism and melanin biosynthesis in appressorium differentiation. We reveal the role of the Pmk1 MAP kinase as a key global regulator of appressorium-associated gene expression. We also provide evidence for differential expression of transporter-encoding gene families and specific high level expression of genes involved in quinate uptake and utilization, consistent with pathogen-mediated perturbation of host metabolism during plant infection. When considered together, these data provide a comprehensive high-resolution analysis of gene expression changes associated with cellular differentiation that will provide a key resource for understanding the biology of rice blast disease.

## Author Summary

The fungus *Magnaporthe oryzae* causes a disease of rice, known as rice blast. Half the world's population depends on rice as a staple food source and rice blast disease destroys 18% of the rice harvest annually. It is therefore important to develop methods to control blast as a means of ensuring global food security. The rice blast fungus spreads rapidly from infected to uninfected plants using a spore known as a conidium. When a conidium lands on the surface of a rice leaf, it develops a specialised structure called an appressorium which is used to penetrate the tough outer cuticle of the rice leaf, enabling the fungus to enter plant tissue. In this study, we have used new sequencing technologies to identify genes that are actively expressed during appressorium formation by looking at relative levels of their transcripts. We have also compared levels of gene expression in a wild-type strain of the fungus to a mutant that is unable to make appressoria and therefore cannot infect plants. The study has enabled us to identify key metabolic processes that are activated during appressorium formation and to understand how fungal metabolism and physiology are dramatically altered during infection-related development.

## Introduction

The ascomycete fungus *Magnaporthe oryzae* is the causal agent of rice-blast disease, which can destroy up to 18% of the annual rice harvest [1]. Because more than half of the global population depends on rice as a staple food crop, rice blast disease represents a significant factor that impacts upon global food security [2]. The genetic tractability of the fungus and availability of a genome sequence also make the organism an excellent experimental model for the study of plant pathogenesis [3].

In common with many plant pathogenic fungi, including rusts and powdery mildews, *M. oryzae* enters its host plant using a specialised infection structure known as an appressorium [1]. Upon landing on a rice leaf, the three-celled asexual spore (called a conidium) germinates, producing a germ tube from one of the apical cells. The end of the germ tube soon swells to form a dome-shaped appressorium, which becomes melanised as it matures [4]. Accumulation of glycerol in the developing appressorium leads to an influx of water by osmosis and the consequent development of hydrostatic turgor of up to 8 MPa [5]. Such high pressure enables the fungus to penetrate the plant cuticle and cell wall by physical force and enter underlying epidermal cells.

Differentiation of functional appressoria is tightly linked to genetic regulation of the cell-cycle. A DNA replication-dependent checkpoint, for instance, is essential for initiation of appressorium formation [6] and entry into mitosis is a pre-requisite for development of a functional appressorium [7]. One of the daughter nuclei from the single mitotic division, which occurs prior to appressorium development, migrates into the developing appressorium, after which septation occurs, separating appressorium from germ tube [8]. The remaining daughter nucleus migrates back to the conidium, which eventually collapses and dies due to infection-associated autophagy [7], [9].

Appressorium formation by *M. oryzae* can be studied away from living plants on artificial, hydrophobic surfaces. Along with the development of methods for routinely performing targeted gene deletions and replacements, this has enabled discovery of important signalling pathways involved in appressorium development, including both cyclic-AMP dependent and mitogen-activated protein (MAP) kinase pathways [10], [11]. Central to appressorium development is the Pmk1 MAP kinase pathway [1], [12], composed of a MAP kinase Pmk1, activated by a MAP kinase kinase (MAPKK) Mst1, which in turn is activated by the Mst11 MAPKK kinase (MAPKKK). The pathway is regulated by the Mgb beta-subunit of a heterotrimeric G-protein and the recently described Msb2 and Sho1 upstream activators [13]. Mutant strains of *M. oryzae*, in which Pmk1 has been deleted, are unable to develop appressoria or grow invasively *in planta*, even when inoculated directly into wounded plant tissue, although growth in axenic culture is largely unaffected [11], [12], [14]. Appressorium formation in *M. oryzae* occurs under conditions where there are no exogenous nutrients available and, therefore, formation of the appressorium and synthesis of large quantities of glycerol involves mobilisation of compounds stored in the conidium. Rapid Pmk1-dependent mobilisation of lipids and glycogen occurs during appressorium development [14], accompanied by an increase in triacylglycerol lipase activity, which liberates glycerol from storage lipids. Fatty acid beta-oxidation has also been shown to be important for appressorium formation, in addition to the glyoxylate cycle, to enable utilization of acetyl-CoA through gluconeogenesis [15], [16]. The importance of the acetyl-CoA pool during appressorium formation is highlighted by the fact that mutants impaired in carnitine acetyl transferase activity are non-pathogenic [17], [18]. Acetyl-CoA is, for instance, needed for synthesis of melanin, cell wall chitin and glucans, as well as potentially being used to synthesise glycerol, and may therefore be pivotal to biosynthetic pathways essential for appressorium function [14], [19].

In order to define the reservoir of gene functions needed for appressorium-mediated plant infection by *M. oryzae*, a systematic analysis of the global patterns of transcriptional activity is necessary. Previous studies have begun to examine this problem by using microarray analysis or, alternatively, massively parallel signature sequencing (MPSS) and serial analysis of gene expression (SAGE), using Sanger sequencing. Each of these studies has, however, focused on only a restricted set of conditions. Donofrio and co-workers [20] demonstrated that a number of known genes involved in pathogenicity were up-regulated under nitrogen starvation when transcripts were analyzed by oligonucleotide-based microarrays, following growth under nitrogen-limiting conditions. However, no direct observations of transcript abundance were made during appressorium development. Gowda and colleagues [21] compared transcript abundance in samples grown as mycelial cultures, or following appressorium development, but included only a single time-point (24 h), by which time the appressorium is fully developed and developmental dynamics are complete. Transcriptional changes have also been compared on inductive and non-inductive surfaces, as well as following addition of exogenous cAMP [22]. However, only two developmental time points were chosen, seven and twelve hours after spore germination. Most recently, a microarray study compared gene expression levels in *M. oryzae* mycelium grown under different stress conditions with those of the fungus growing *in planta* [23]. The authors concluded that during invasive growth *M. oryzae* may grow under conditions of nutrient starvation, consistent with earlier studies that made similar conclusions [24], [25]. However, each of these data sets, while providing valuable information, presented expression patterns for only a sub-set of *M. oryzae* genes and a restricted set of time-points.

In this study we have taken advantage of the utility of next generation-sequencing (NGS) to perform a comprehensive analysis of gene expression throughout appressorium development in *M. oryzae* at much greater sensitivity than was hitherto possible using either microarray or tag-based approaches. There are two evolving methods to apply NGS sequencing to measure gene expression changes, RNA-Seq, in which whole transcripts are sequenced [26] and tag-based methods such as Digital Gene Expression (DGE) and High Throughput (HT)-SuperSAGE [27]. We employed both RNA-Seq and HT-SuperSAGE and found that HT-SuperSAGE provides data that corresponds well with RNA-Seq from the same tissues, but at a much higher throughput and reduced cost. Subsequently, we used HT-SuperSAGE to analyse global patterns of gene expression during appressorium development of *M. oryzae*. Here, we present transcript profiles of 10,591 genes, 96% of the total predicted genes of *M. oryzae*, thus providing the most complete coverage of the transcriptome in *M. oryzae* published studies. This has enabled identification of genes that are highly expressed at specific stages of appressorium development. We have used these data to compile a publicly accessible database (http://cogeme.ex.ac.uk/supersage/) as part of the COGEME suite of databases [28] to provide expression values for any specified gene in the *M. oryzae* genome during appressorium morphogenesis. We present the most significant changes in gene expression and reveal major metabolic and physiological changes associated with infection-related development by the rice blast fungus.

## Results

### Comparison between RNA-Seq and HT-SuperSAGE

Two alternative high-throughput methods exist for generation of transcriptomic data. In RNA-Seq, sequences are derived from total RNA, reverse transcribed to cDNA, fragmented and sequenced using next-generation DNA sequencing (NGS) technology [26]. The short reads produced by NGS sequencing are then assembled after alignment to a reference genome and in this way the complete sequence of each transcribed gene can be obtained, allowing identification of splice sites, un-translated regions, alternatively spliced transcripts and complete gene coding sequences. It is also possible to use these data to quantify abundance of each transcript in the cDNA library by calculating the frequency of short reads that align to each gene [29]. These values are normalised to take account of differing lengths of genes and the total number of short reads obtained from each library and are generally expressed as fragments per kilobase of exon, per million fragments mapped (FPKM). An alternative method for quantifying levels of transcript abundance is HT-SuperSAGE [27]. In this method cDNA is prepared from each tissue sample. Twenty-six base sequence tags are then independently generated from each transcript in these libraries and NGS technology used to sequence tags [27]. Sequence tags are aligned back to a reference genome and the number of tags from each gene calculated to provide a measure of gene expression. Values are normalised to take into account the total number of tags sequenced from each library and are typically expressed as a fraction of the total number of sequenced tags (tags per million or TPM). The advantage of HT-SuperSAGE over RNA-Seq for the analysis of transcript abundance is that a lower depth of sequencing is required. It has been estimated, for example, that to achieve 90% coverage of the human transcriptome, 40 million reads would be required using RNA-Seq, compared to less than 5 million reads using HT-SuperSAGE [30]. This makes it feasible to run multiple HT-SuperSAGE samples simultaneously, using unique 4-base bar codes to distinguish between tags from different samples and reducing costs further [27].

In order to make a comparison between these two techniques, we analysed transcript abundance in *M. oryzae* mycelium grown in complete medium (CM), compared to *M. oryzae* grown in glucose minimal medium (MM) for 36 h. Each RNA-Seq sample required one lane in an Illumina flowcell, whereas four HT-SuperSAGE samples were analysed per lane. The number of individual transcripts identified using each of these two techniques was very similar; 9,985 using RNA-Seq and 9,989 using HT-SuperSAGE from at least one of the two samples of mycelium (CM and MM). A Pearson correlation coefficient of 0.57 was recorded when data from both methods were compared. A previous study comparing quantitative gene expression values in appressorium and mycelium from *M. oryzae* using MPSS, robust-long SAGE (RL-SAGE) and microarray analyses produced pair-wise correlation coefficients ranging from only 0.068 to 0.59 using unfiltered data sets. These correlation coefficients could be increased by filtering data sets either by removing genes with low levels of expression or removing outliers [22]. The use of NGS methodologies overcame such limitations. Therefore the two methods produced very similar levels of sensitivity, but HT-SuperSAGE proved much more cost effective.

### Changes in gene expression during appressorium development

**Figure 1:**
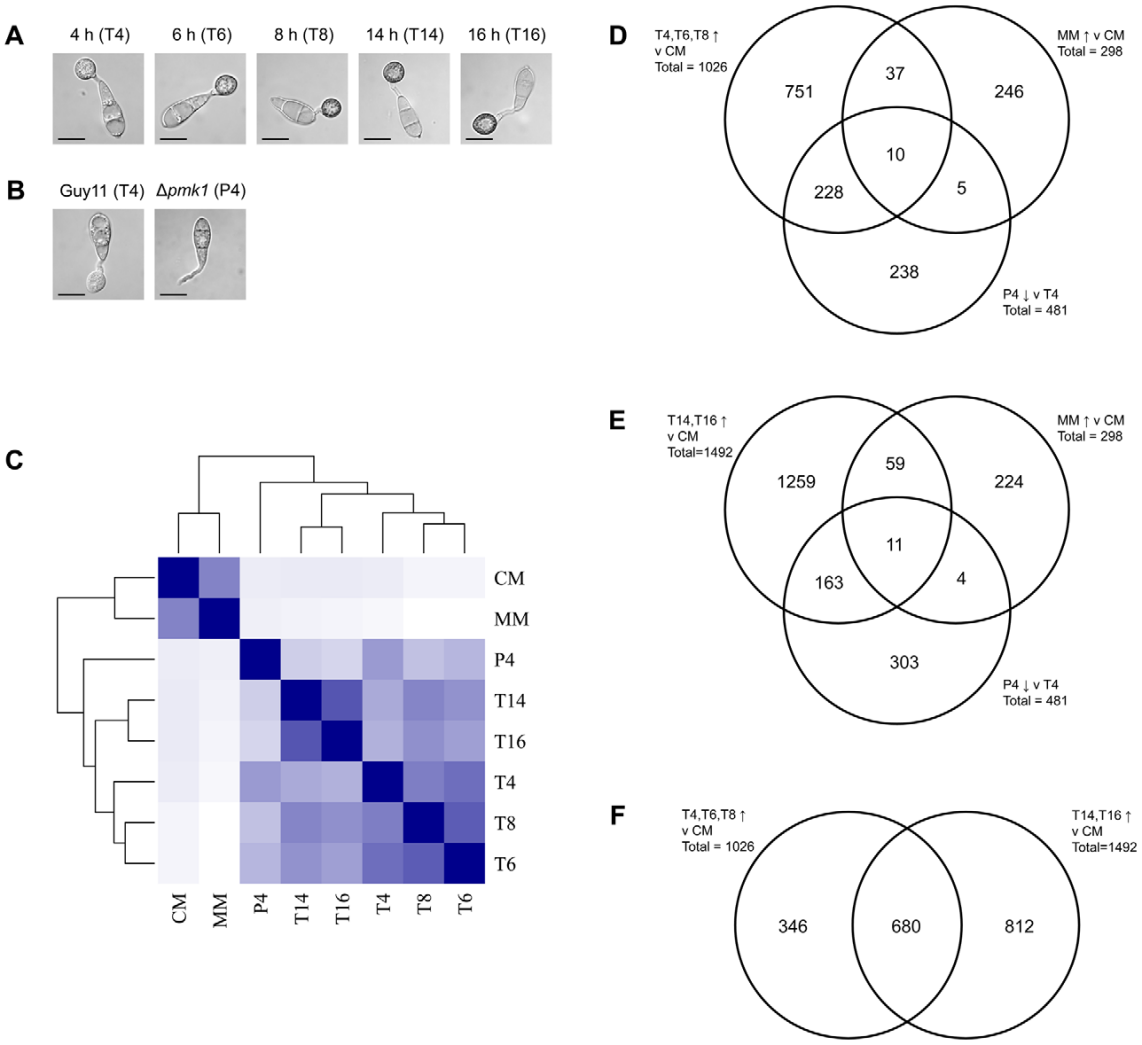
Overall comparison of HT-SuperSAGE datasets. **A**. Micrographs showing appressorium development at time-points used for HT-SuperSAGE analysis (scale bar = 10 µm). **B**. Micrographs comparing appresorium development at 4 h in wild-type Guy11 and Δ*pmk1* mutant backgrounds. **C**. Heatmap showing Euclidean distances between HT-SuperSAGE samples as calculated from a variance-stabilising transformation of the total count data. The darker the colour, the closer the two datasets are together. T4, T6, T8, T14 and T16 are time-points during appressorium development in Guy11, P4 is appresorium development at 4 in a Δ*pmk1* mutant. CM and MM are samples from Guy11 mycelium grown in complete medium and minimal medium respectively. **D**. Venn diagram illustrating overlaps between number of genes that are significantly up-regulated (P-value<0.05) in early appressorium development – 4 h to 8 h (vs mycelium grown in CM), significantly up-regulated (P-value<0.05) in mycelium grown in minimal medium (vs mycelium grown in CM) and significantly down-regulated (P-value<0.05) in a Δ*pmk1* mutant compared to Guy11 after 4 h **E**. Venn diagram illustrating overlaps between number of genes that are significantly up-regulated (P-value<0.05) in late appressorium development – 14 h to 16 h (vs mycelium grown in CM), significantly up-regulated (P-value<0.05) in mycelium grown in minimal medium (vs mycelium grown in CM) and significantly down-regulated (P-value<0.05) in a Δ*pmk1* mutant compared to Guy11 after 4 h. **F**. Venn diagram illustrating overlaps between number of genes that are significantly up-regulated (P-value<0.05) in early appressorium development – 4 h to 8 h (vs mycelium grown in CM) and late appressorium development – 14 h to 16 h (vs mycelium grown in CM).

Having established the sensitivity and accuracy of HT-SuperSAGE, we applied the method to reveal global patterns of gene expression during appressorium development by *M. oryzae*. RNA was extracted from conidia germinated on a hydrophobic glass slide for 4, 6, 8, 14 and 16 h, respectively (Figure 1A). Two replicates for each time-point were taken and HT-SuperSAGE of cDNA was used to measure transcript abundance, representing individual genes at each time point. Time points were chosen to target discrete developmental stages associated with appressorium morphogenesis. Two broad stages can be readily discerned during appressorium formation: a *development* phase from approximately 4 to 8 h after germination, during which the germ tube tip swells forming a characteristic hemispherical shape and cellular components begin to migrate into this nascent appressorium, which subsequently becomes melanin-pigmented, and a *maturation* phase after 8 to 16 h during which the appressorium becomes pressurised due to the increased production of solutes and conidial cells undergo autophagy and cell death [1]. To define major transcriptional changes associated with appressorium development sampling was carried out at 4, 6 and 8 h, while the maturation phase was further monitored at 14 and 16 h. For comparison, HT-SuperSAGE data were generated from mycelium grown in rich complete medium (CM) or under conditions of nutrient limitation in glucose minimal medium (MM). To define further the gene expression profiles specific to appressorium morphogenesis, we analysed a mutant lacking the *PMK1* MAP kinase-encoding gene, which is vital for appressorium formation and pathogenicity [11]. Strains lacking *PMK1* cannot form appressoria and are non-pathogenic even when spores are injected directly into plant tissue [11], [12], [14]. HT-SuperSAGE data was used to compare transcript abundance in germinating conidia 4 h after they were placed on an inductive surface. Figure 1B shows that at this time-point, an appressorium is already developing in Guy11 strain whereas no infection cell development occurs in an isogenic Δ*pmk1* mutant [11].

To assess the overall similarity between data sets, the Euclidean distance was calculated between each sample, using transcript abundance values for all genes. Data sets were clustered based on these distances and a heat map generated (Figure 1C). The results show that data sets generated from mycelium are highly distinctive from transcripts associated with appressorium development. Within appressorium datasets, the Δ*pmk1* mutant showed global patterns of gene expression that formed a separate clade from datasets generated from wild-type *M. oryzae*, suggesting that the *PMK1* MAP kinase affects the expression level of a significant number of genes associated with cellular differentiation. The time-points at 4–8 h also form a separate clade from those at 14 and 16 h, consistent with there being two distinct phases of appressorium development.

We were specifically interested in identifying gene expression patterns during appressorium development, suggesting physiological or signalling pathways important in cellular morphogenesis. As a baseline for comparison, we looked at gene expression during growth of mycelium in rich medium in which no comparable cellular differentiation occurs. A total of 1026 genes were identified that were significantly up-regulated (adjusted P-value< = 0.05) at all time-points between 4 and 8 h when compared to expression in mycelium grown in CM (Table S1). An analysis was performed on this data set to predict functions of each gene identified based on gene ontology (GO) categories using Blast2GO [31]. GO categories over-represented within appressorium differentially-expressed genes were identified using Fisher's exact test (adjusted P-value< = 0.05, Table S1). Over-represented GO categories were consistent with increased carbohydrate metabolism, expression of a large set of glycosyl hydrolases and sugar transporter-encoding genes and induction of secondary metabolic pathways. We also identified genes encoding proteins important in cell cycle control such as the G2-specific protein kinase-encoding gene *NIMA* (MGG_03026) [6], [7], a homologue of cdc14 from *Schizosaccharomyces pombe* (MGG_00757), which is part of the complex that controls septation and cytokinesis [32], a homologue of *tinA* from *Aspergillus nidulans* (MGG_00763), a gene encoding a protein that interacts with *NimA* [33], and homologues of the kinetochore protein-encoding gene Mis14 (MGG_00906) and *sudA* (MGG_04988) from *Aspergillus nidulans* which are involved in chromosome segregation during mitosis [34]. The homologues of cdc14, Mis14, *tinA* and *sudA* all show significant up-regulation during early appressorium development at 4 and 6 h after conidial germination. In fact, the Mis14 homologue is significantly up-regulated throughout appressorium development and the *sudA* homologue is also significantly up-regulated at 8 h. *NIMA* is only significantly up-regulated 8 hours after the start of conidial germination. The data are also consistent with *NIMA* and the homologues of *sudA* and cdc14 being under transcriptional control of the Pmk1 MAP-kinase pathway. Appressorium development in *M. oryzae* is regulated by cell cycle control and temperature sensitive mutants in *NIMA*, for instance, are unable to produce appressoria at restrictive temperatures [6], [7]. We also found evidence for co-regulation and differential expression of autophagy-associated genes (Figure S1). Hierarchical clustering grouped twelve of these genes into a clade showing up-regulation during early stages of appressorium formation. Infection-associated autophagy is necessary for appressorium function and deletion of any one of sixteen genes involved in non-selective macroautophagy in *M. oryzae* leads to loss of pathogenicity [9]. A total of 1492 genes were identified as significantly up-regulated (adjusted P-value< = 0.05) at both 14 h and 16 h, when compared to mycelium grown in CM (Table S2). GO categories over-represented in this dataset included those involved in carbohydrate metabolism, specifically the large and diverse set of glycosyl hydrolases encoded by *M. oryzae*, transmembrane transport, particularly of sugars, developmental processes such as cell wall biogenesis and the response to different external stimuli (including, for example, cAMP). In total, 481 genes were identified showing significantly lower levels of expression in a Δ*pmk1* mutant when compared to the wild-type at 4 h (adjusted P-value< = 0.05) (Table S3). These genes are therefore likely to be positively regulated by the presence of an active *PMK1* MAP kinase pathway. GO categories over-represented in this dataset were those specifically involved in response to exogenous stimuli, including two CFEM-domain containing receptor proteins (Table S3) and a large set of 15 transporter-encoding genes, as well as 16 putative transcription factor-encoding genes differentially expressed as a consequence of loss of *PMK1*.

During formation of the appressorium *in vitro*, the germinating conidium is under nutrient limited conditions and therefore a number of genes may be up-regulated solely as a response to starvation stress. To identify genes up-regulated by nutrient limitation, expression levels were compared between *M. oryzae* mycelium grown in glucose minimal medium (MM) and complete medium (CM). In this way 298 genes were identified that were up-regulated in mycelium grown in MM compared to CM (Table S4). The GO categories over-represented in the data set were involved in transmembrane transport, redox control and developmental processes. The Venn diagrams in Figure 1 provide an illustration of the overlaps between each distinct transcriptionally-defined gene set. For example, of the 481 genes that are down-regulated in a Δ*pmk1* mutant, nearly half (238) are also up-regulated during the early stages (4–8 hours) of appressorium development. A smaller number of Δ*pmk1* down-regulated genes (174) are differentially regulated during the later stages of appressorium development (14–16 hours). The overlap between genes up-regulated by nutrient limitation and those up-regulated during appressorium development is, however, much lower (15% and 24% of the MM up-regulated genes are also up-regulated during early and late appressorium development, respectively). This suggests that nutrient limitation acts as an inducing signal for only a small proportion of genes up-regulated during appressorium development.

### Generation of acetyl-CoA during development of appressoria

**Figure 2:**
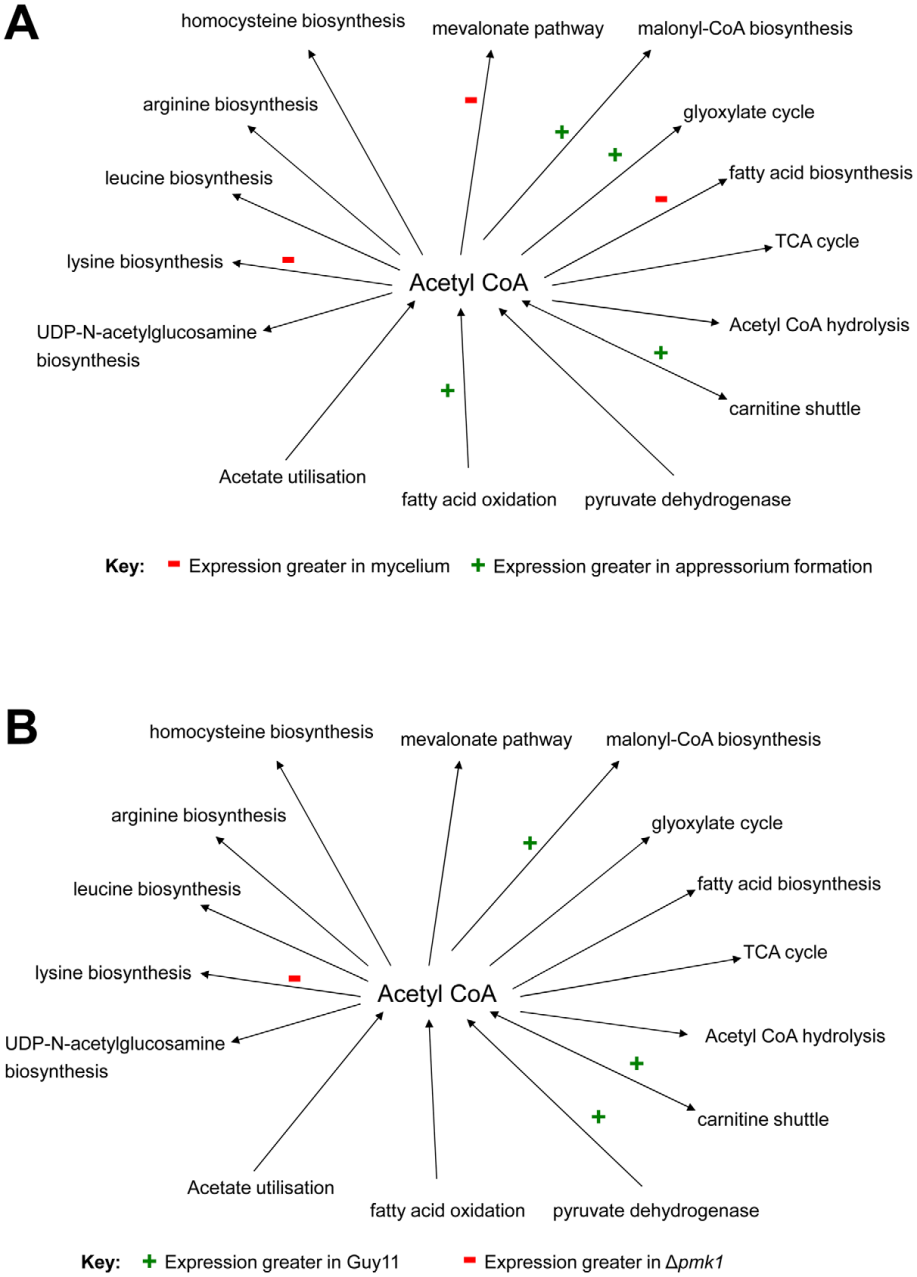
Expression level of genes encoding enzymes that produce or utilise acetyl-CoA. The diagrams illustrate metabolic pathways that produce (arrow pointing towards acetyl-CoA) or utilise (arrow pointing away from acetyl-CoA) acetyl-CoA. Transcript abundance was compared in those genes from each pathway that encode enzymes that directly utilise or produce acetyl-CoA **A**. Guy11 germinating conidia (4 h) and Guy11 mycelium grown in complete medium. **B**. Δ*pmk1* germinating conidia (4 h) and Guy11 germinating conidia (4 h). The diagrams also show which pathways contain genes significantly more highly expressed in one condition as compared to the other (see key for each diagram). These are based on adjusted P-value< = 0.05 for at least one of the genes in each pathway.

As a consequence of the importance of the acetyl-CoA biosynthesis and metabolism to appressorium development [14]–[19], we next selected 31 genes encoding enzymes that putatively utilise or produce acetyl-CoA. Table S5 shows the HT-SuperSAGE data for this population of genes during appressorium development in Guy11, in mycelium grown in both CM and MM, and in germinating conidia of a Δ*pmk1* mutant at 4 h after being placed on an inductive surface. The results are summarised by each pathway in Figure 2. The predicted pathways in which the enzymes that utilise/produce acetyl-CoA have a higher or lower expression (adjusted P-value< = 0.05) during appressorium formation (at 4 h) when compared to mycelial growth, is shown in Figure 2A. Genes encoding enzymes from the pathway that oxidises fatty acids to produce acetyl-CoA, for example, show increased expression during appressorium formation, as do carnitine acetyl transferases, which transport acetyl-CoA between sub-cellular compartments. The enzyme acetyl-CoA carboxylase, that synthesises malonyl-CoA from acetyl-CoA [35], shows increased expression during appressorium formation, while acetyl-CoA-utilising enzymes in the fatty acid, mevalonate and lysine biosynthesis pathways all showed lower expression. Figure 2B shows pathways in which the enzymes that utilise/produce acetyl-CoA have a higher or lower expression (adjusted P-value< = 0.05) during appressorium formation (at 4 h) in Guy11 compared to a Δ*pmk1* mutant. Acetyl-CoA carboxylase, glyoxylate cycle genes, pyruvate dehydrogenase and carnitine acetyl transferase genes were all reduced in expression in a Δ*pmk1* mutant when compared to the isogenic Guy11. In contrast, genes encoding acetyl-CoA producing/utilising enzymes involved in lysine biosynthesis showed greater expression in a Δ*pmk1* mutant. Overall, these results confirm that acetyl-CoA plays a central role in appressorium morphogenesis, being mainly produced from beta-oxidation of fatty acids and then used for biosynthesis of malonyl-CoA or the glyoxylate shunt. Only acetyl-CoA carboxylase and the carnitine acetyl-transferases appear to be differentially regulated as a consequence of the presence of the Pmk1 MAP-kinase pathway. Peroxisomal beta-oxidation of fatty acids [16] and subsequent transport of acetyl-CoA out of the peroxisome by carnitine acetyltransferase have both been shown to be necessary for formation of functional appressoria [17]. Appressorium formation is furthermore delayed in strains in which the glyoxylate cycle enzyme isocitrate lyase is deleted [15].

### Malonyl-CoA utilisation and control of appressorium-specific secondary metabolism

**Figure 3:**
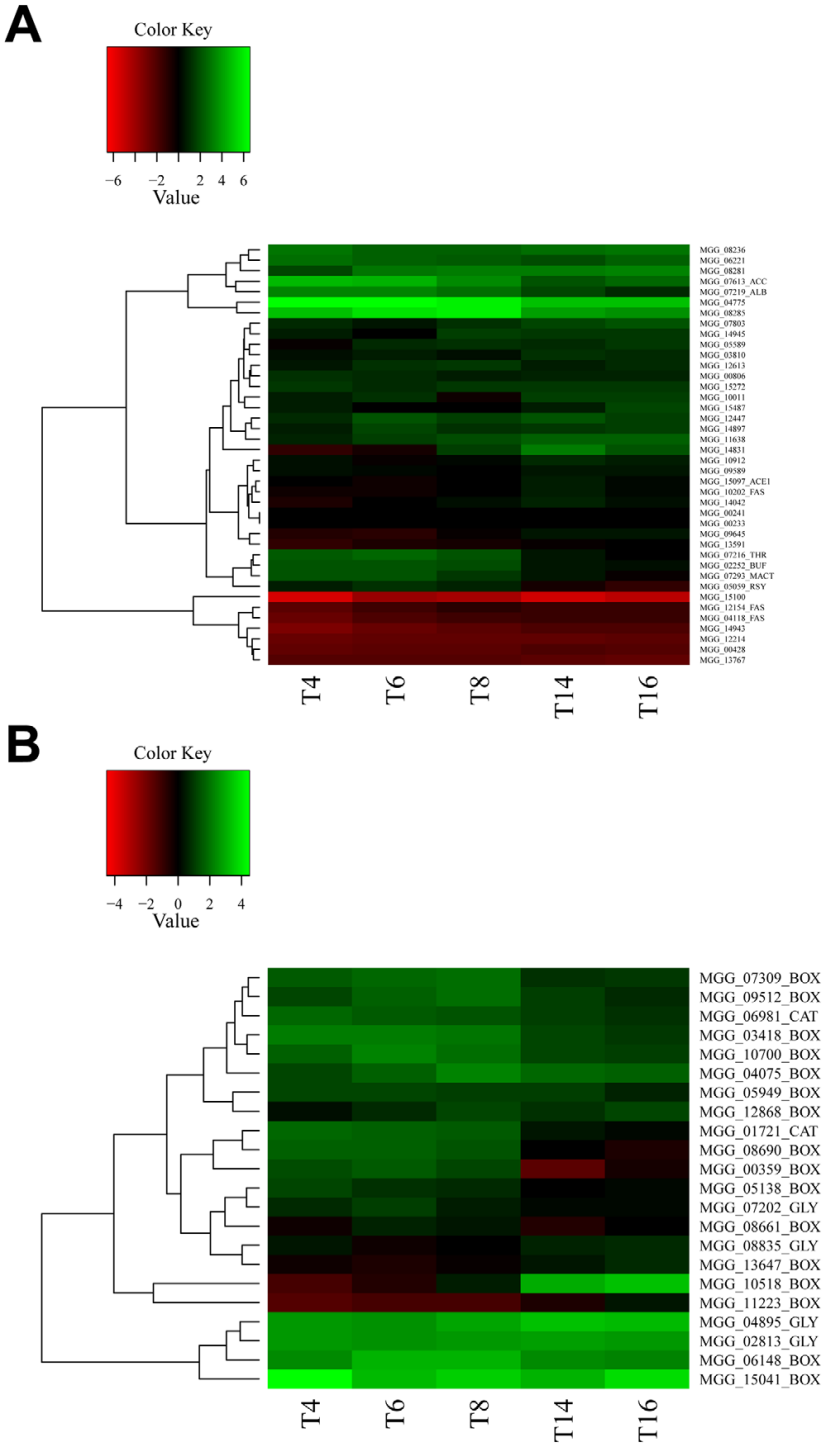
Heatmaps showing levels of transcript abundance during time course of appressorium development. Levels of expression are represented as moderated log_2_ ratio of transcript abundance compared to mycelium grown in complete medium. Values are from 4 h (T4) to 16 h (T16) after conidia were placed on a hydrophobic surface. Genes showing similar patterns of gene expression are clustered. **A**. Genes encoding enzymes from pathways that utilise malonyl-CoA. Specifically labelled are acetyl-CoA carboxylase (ACC), malonyl CoA-acyl carrier protein trans-acylase (MACT), fatty acid synthase (FAS), components of melanin biosynthesis pathway (ALB, BUF, RSY, THR) and the PKS-NRPS hybrid ACE1. **B**. Genes encoding enzymes involved in β-oxidation of fatty acids and the glyoxylate cycle. Genes are labelled according to pathways: fatty acid β-oxidation (BOX), glyoxylate cycle (GLY) and carnitine acetyl-transferases (CAT).

To follow the likely fate of acetyl CoA during appressorium differentiation we next analysed expression of genes associated with malonyl CoA synthesis and metabolism. Acetyl-CoA carboxylase is highly up-regulated during appressorium development and differentially regulated by the presence of *PMK1*. This enzyme synthesises malonyl-CoA from acetyl-CoA [35]. Malonyl-CoA is used as a substrate by both polyketide and fatty acid synthases. In order to determine the likely fate of malonyl CoA during fatty acid metabolism, HT-SuperSAGE data were analysed for polyketide synthase expression, as well as genes involved in fatty acid biosynthesis (Table S6). Nine polyketide synthase and four hybrid polyketide synthase / non-ribosomal peptide synthases were significantly up-regulated (adjusted P-value< = 0.05) in at least one time-point during appressorium development. These included *ALB1*, which encodes a polyketide synthase that catalyses the first step in melanin biosynthesis [36]. Consistent with the importance of malonyl-CoA synthesis during appressorium formation, is the up-regulation of the malonyl CoA-acyl carrier protein transacylase gene, which transfers malonyl-CoA thioesters from solution to fatty acid synthases or polyketide synthases [37]. Expression of malonyl-CoA utilisation genes was visualised as a heat map (Figure 3A), created using moderated log_2_-fold changes of transcript abundance during appressorium development, compared to expression in mycelium. Genes showing similar patterns of expression were grouped together by hierarchical clustering. The gene encoding acetyl-CoA carboxylase, for example, clusters together with the gene encoding *ALB1*, while the rest of the melanin biosynthesis pathway genes cluster with the gene encoding malonyl CoA-acyl carrier protein transacylase (Figure 3). This suggests a strong link between melanin biosynthesis pathway and malonyl-CoA metabolism. Differential co-ordinated expression of fatty acid synthases, putatively associated with generation of very long chain branched fatty acids, such as mycocerosic acid (MGG 04775 and MGG 08285), was also observed along with a large clade of 13 co-ordinately-regulated polyketide synthases and a separate clade of hybrid polyketide synthase, non-ribosomal peptide synthetases, including the *ACE1* avirulence gene [38].

### Differential expression of lipid mobilisation-associated genes during appressorium development

To investigate gene expression associated with appressorial mobilisation of lipids [14] and their subsequent metabolism [16], we next selected the entire set of predicted lipid metabolic genes. Table S7 shows transcriptional profiling data for genes encoding enzymes from these pathways and Figure 3B shows a heat map to illustrate some of the principal changes in appressorium-associated expression. Most genes involved in beta-oxidation of fatty acids are significantly up-regulated during appressorium development, with those encoding the multifunctional beta-oxidation enzyme *MFP1* (MGG_6148) and an acyl-CoA dehydrogenase (MGG_15041) being most up-regulated. In a heat map these two genes cluster with two other genes encoding specific enzymes of the glyoxylate shunt, such as isocitrate lyase (MGG_04895) and malate synthase (MGG_02813), consistent with activation of the pathway during appressorium maturation (Figure 3B). The cytosolic isozyme of malate dehydrogenase (MGG_08835) does not show such high relative levels of expression during appressorium development, but is also involved in shuttling oxaloacetate from mitochondria to the cytosol [39] and may therefore not be specifically induced during appressorium formation. The glyoxylate cycle also requires a non-mitochondrial citrate synthase, but analysis of the *M. oryzae* genome shows only one putative citrate synthase (MGG_07202) and one methylcitrate synthase-encoding gene (MGG_02617), likely involved in propionate metabolism [40], both of which are predicted to be mitochondrial. It may be that different transcripts encoding isozymes of citrate synthase with different locations can be synthesised from the same gene, as observed for NADP-dependent isocitrate dehydrogenases from *Aspergillus nidulans* [41]. If this is the case, it is not surprising that the citrate synthase gene does not show the same pattern of expression as glyoxylate cycle-specific genes. The glyoxylate cycle enables acetyl-CoA produced by beta-oxidation of fatty acids to be fed into gluconeogenesis allowing glycerol, glucans and chitin to be synthesised [15]. Intracellular transport of acetyl-CoA produced during beta-oxidation by the peroxisomal carnitine acetyltransferase *PTH*2 is necessary for appressorium function [17], [18]. HT-SuperSAGE revealed that *PTH2* (MGG_01721) is highly expressed between 4–8 h and likely to be under the control of the Pmk1 MAP kinase pathway.

**Figure 4:**
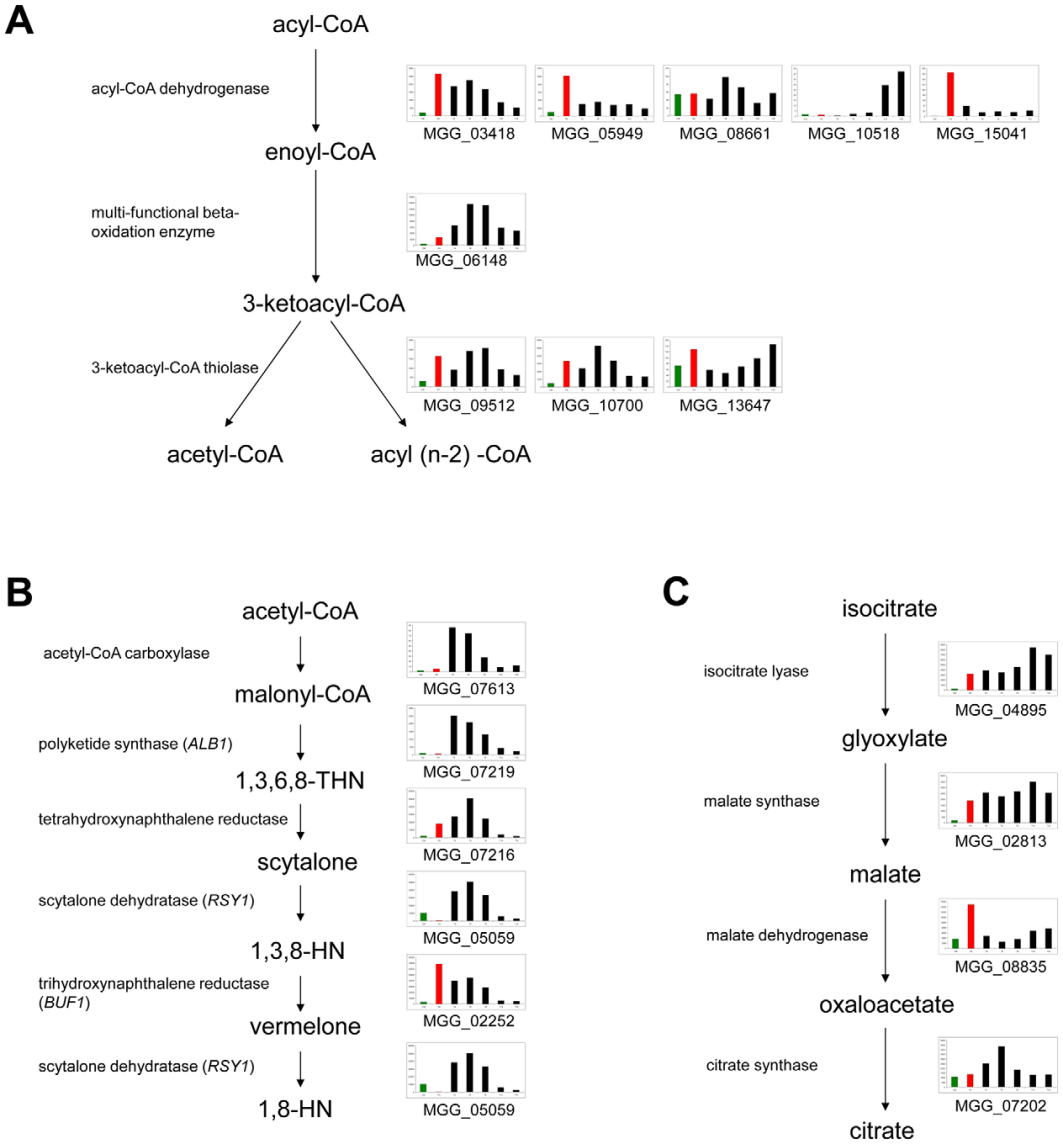
Expression of genes encoding enzymes from three metabolic pathways that affect the pool of acetyl-CoA. **A**. The β-oxidation pathway, **B**. melanin biosynthesis, **C**. the glyoxylate shunt. For each enzyme, bar graphs show abundance of transcripts encoding this gene in: Guy11 mycelium grown in complete medium (green bar), Δ*pmk1* mutant conidia left to germinate for 4 h (red bar), Guy11 time course of appresorium development (4 h, 6 h, 8 h, 14 h, 16 h – black bars left to right).

Our data therefore independently confirm that fatty acid beta-oxidation, melanin biosynthesis and the glyoxylate shunt are pivotal processes during appressorium development, consistent with gene functional studies [1], marking major changes in metabolism that are necessary for infection cells to develop and function correctly. Figure 4 shows expression patterns of the key enzymes in each of these pathways. Figure 4A shows the core peroxisomal fatty acid beta-oxidation pathway in which nearly all genes show higher levels of expression during appressorium development (black bars) than in mycelium grown axenically. Only the multi-functional beta-oxidation enzyme showed reduced expression in a Δ*pmk1* mutant (red bar) compared to the wild-type, suggesting that it might be controlled by the Pmk1 MAP kinase pathway.

Expression profiles of genes encoding enzymes of the dihydroxynaphthalene (DHN) melanin biosynthesis pathway are shown in Figure 4B. They all show very similar patterns of expression, with high levels at 4 and 6 h when the appressorium is developing, but reducing from 8 h onwards at the onset of maturation. Expression profiles of genes encoding enzymes of the glyoxylate cycle are shown in Figure 4C. Three of the four enzymes showed higher level of expression throughout appressorium development and are also significantly reduced in expression in a Δ*pmk1* mutant compared to the wild-type Guy11. The gene encoding cytosolic malate dehydrogenase, however, showed a different expression profile which may be due to the fact that cytosolic malate dehydrogenase is a component of both the glyoxylate cycle and the malate-aspartate shuttle [39]. The latter pathway is responsible for translocating reducing equivalents in the form of NADH produced by glycolysis across the mitochondrial inner membrane for oxidative phosphorylation [39]. This pathway is likely to be active during both mycelial growth and development of the appressorium, and it is therefore not surprising that the malate dehydrogenase gene is highly expressed in both tissue types.

### Differential expression of transmembrane transporter-encoding genes during appressorium morphogenesis by *M. oryzae*

Gene expression profiles within mature appressoria at the point of penetration peg emergence would be expected to predict the likely repertoire of gene functions associated with initial growth in plant tissue and may therefore be valuable in identifying the principal substrates used by the fungus during its growth in plant cells. Phytopathogenic fungi are osmotrophic micro-organisms reliant on the secretion of a broad repertoire of depolymerising enzymes and a range of transporters to acquire nutrients from their host, as well as to export toxins and remove anti-fungal compounds produced by the plant. We identified expression data for 206 genes encoding secreted enzymes that breakdown carbohydrates (Table S8). Of these, 72 were significantly up-regulated during appressorium development, and only 30 down-regulated. The classes of enzymes that showed differential up-regulation of expression during appressorium development included many that potentially degrade components of the plant cell wall, for example, cutinases, endo-1,4-beta-xylanases, a polygalacturonase, cellulases, a rhamnogalacturonan acetylesterase and alpha-L-arabinofuranosidases. In addition, genes encoding enzymes involved in the extensive fungal cell wall remodelling that goes on during appressorium development, for example, chitinases and beta-hexosaminidases were differentially expressed. In parallel, a search for *M. oryzae* proteins with Pfam motif PF00083, a signature of saccharide (and other) transporters of the major facilitator superfamily (MFS) of membrane transporters, identified 71 genes (Table S9), of which 29 were differentially expressed during appressorium formation. When considered together, these data suggest that the maturing appressorium expresses genes leading to rapid secretion of a large repertoire of enzymes to break down plant oligosaccharides and a range of other plant cellular components into monosaccharides and simple monomers, with expression of cognate transporters to import these products into the invading pathogen.

Among sugar transporter-encoding genes we noted a family of four putative quinate permease genes (MGG_07779, MGG_14136, MGG_09778 and MGG_04225), differentially expressed at all stages of appressoria formation. Only one of the quinate permeases, MGG_09778 is expressed in a Δ*pmk1* mutant at 4 h post germination. Four other quinate permeases were also detected at levels not significantly different from mycelium growing in CM, suggesting that distinct families of the transporter may be deployed in mycelial growth and plant infection.

**Figure 5:**
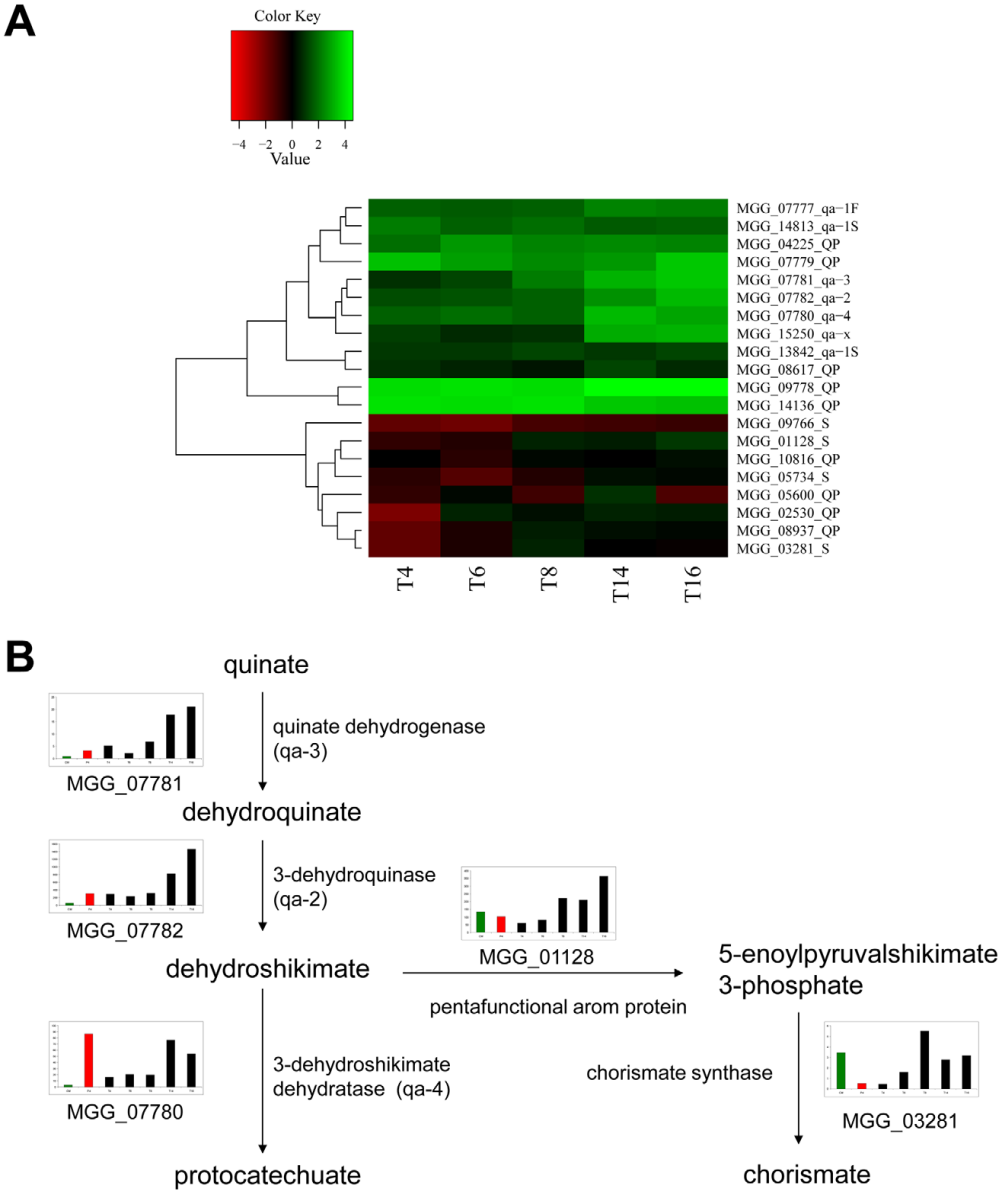
Levels of transcript abundance from genes involved in quinate metabolism. **A**. Heatmap showing levels of transcript abundance during time course of appressorium development. Levels of expression are represented as moderated log_2_ ratio of transcript abundance as compared to mycelium grown in complete medium. Values are from 4 h (T4) to 16 h (T16) after conidia were incubated on a hydrophobic surface. Genes showing similar patterns of gene expression have been clustered. Homologues of genes involved in quinate metabolism in *Neurospora crassa* are labelled: quinate activator (qa-1F), quinate repressor (qa-1S), 3-dehydroquinase (qa-2), Quinate dehydrogenase (qa-3), 3-dehydroshikimate dehydratase (qa-4), function unknown (qa-x), quinate permeases (QP), shikimate pathway (S). **B**. Expression of genes involved in quinate metabolism. For each enzyme, bar graphs show abundance of transcripts encoding the gene in: Guy11 mycelium grown in complete medium (green bar), Δ*pmk1* mutant conidia left to germinate for 4 h (red bar), a Guy11 time course of appressorium development (4 h, 6 h, 8 h, 14 h, 16 h – black bars left to right).

Interestingly, quinate can serve as sole carbon source for several fungi and the pathway has been studied in detail in *Neurospora crassa* [42] and *Aspergillus nidulans* [43]. Quinic acid is a cyclic polyol and an abundant carbon source that can account for up to 10% of decaying leaf litter [43]. Interestingly, a recent metabolite profiling study of rice blast-infected leaves noted an increase in quinate at early stages of *M. oryzae* infection and suggested that the invading fungus may modulate host metabolism to divert metabolites, such as dehyroquinate and dehydroshikimate that are shared between the quinate and shikimate pathways to quinate production, thereby reducing defensive phenylpropanoid production through the shikimate pathway [44]. Quinate produced in such a way could serve as a very good source of carbon for *M. oryzae*, which is less readily utilizable by the rice host. To test this idea, we investigated whether *M. oryzae* genes encoding enzymes required for quinate metabolism were also differentially expressed in developing appressoria (Figure 5, Table S10). We found that quinate dehydrogenase, 3-dehydroquinase and 3-dehydroshikimate dehydratase are also differentially expressed during appressorium maturation. Quinate metabolism is also subject to catabolite repression, is induced by quinic acid, and co-regulated at the transcriptional level by activator (MGG07777) and repressor proteins (MGG1842, MGG14813), which we also found to be differentially expressed during appressorium formation (Table S10). Furthermore, expression of genes involved in the anabolic shikimate pathway encoding the penta-functional AROM protein, chorismate synthase and 3-deoxy-D-arbinoheptulosonate-7-phosphate synthase were not significantly up- regulated during appressorium formation, providing further evidence for the quinate metabolic pathway being active. Taken together, these observations strongly suggest a role for quinate as a carbon source for *M. oryzae* during plant infection. Protocatechuic acid, the end product of quinate pathway may be further degraded via the β-ketoadipate pathway into succinate and acetyl CoA and enter the TCA cycle [45]. However, in fungi the pathway has only been studied biochemically and only one gene for beta-carboxy-cis,cis-enzyme has been cloned from *Neurospora crassa* [46]. Interestingly, the *M. oryzae* homolog of this gene, MGG 1335 is significantly expressed during appressorium development at 6 h.

In contrast to the large number of sugar transporters up-regulated during appressorium formation, only 4 of the 38 putative organic acid transporter genes (GO annotation; includes all genes containing Pfam motif PF000324 for amino acid permeases) were significantly up-regulated at any time point during appressorium formation (Table S11). Of the four, only one, a proline-specific permease (MGG_02899), was specifically expressed in appressoria at all time points. Furthermore, ten of the organic acid transporters were down-regulated in appressoria during both development (4–8 hrs) and maturation phases (14–16 hrs). This group included proline and lysine specific permeases (MGG_04216, MGG_08129, MGG_14937), GABA permease (MGG_14115), another excitatory amino acid transporter (MGG_07639) and an orthologue of isp4 from *Schizosaccharomyces pombe*, an oligopeptide transporter, which is up-regulated in fission yeast in response to nitrogen starvation [47]. Overall, our observations suggest that amino acid uptake is unlikely to be a significant process during appressorium development and the initial stages of plant infection.

The other major group of transporters we investigated were the ABC transporters, MFS transporters and multidrug and toxin extrusion (MATE) family of transporters, that are often annotated as drug transporters (see, for example, Blast2Go or the Magnaporthe genome database at http://www.broadinstitute.org/annotation/fungi/magnaporthe/). Ninety one such transporter genes were analyzed for expression during appressorium development (Table S12). We found that 35 putative drug transporter genes were significantly up-regulated during appressorium development, and 18 down-regulated. Only three of the transporters were also up-regulated in mycelium growing on MM and another two in a Δ*pmk1* mutant, indicating that the majority of these transporters (31) are expressed specifically during appressorium function and may be deployed to deliver secondary metabolites into the host or to protect the fungus from plant defence compounds during pathogenesis. Six of the drug transporters (MGG_11025, MGG_13762, MGG_09976, MGG_03557, MGG_10336 and MGG_10534) were significantly up-regulated at all stages of appressorium development. The MGG_13762 gene encodes the previously characterised *ABC3* transporter gene, which is required for host penetration [48]. Two other previously reported ABC transporters, *ABC1* (MGG_13624) [49] and *ABC4* (MGG_00937) [50] implicated as pathogenicity factors in *M. oryzae*, were also differentially expressed in appressoria. Consistent with the differential expression of putative efflux pumps, 18 key secondary metabolic pathway enzymes are also significantly up-regulated in at least one time-point during appressorium development, consistent with an overall increase in secondary metabolite synthesis during appressorium development.

### Analysis of expression of hydrophobins and hydrophobic surface binding proteins

Fungal hydrophobins are small, hydrophobic proteins secreted by fungi and are essential for the formation of aerial structures and mediate the attachment of the fungus to hydrophobic surfaces such as the rice leaf surface [51]. The class I hydrophobin *MPG1* [24], [25] and the class II hydrophobin *MHP1* [52] are both required for full pathogenicity of *M. oryzae*. Two other class II hydrophobin encoding genes have also been discovered in the genome of *M. oryzae* [52]. Another secreted fungal protein that binds to hydrophobic surfaces is encoded by *HsbA* from *Aspergillus oryzae* [53]. This secreted protein binds to artificial polybutylene succinate-co-adipate (PBSA) hydrophobic surfaces and has been shown to recruit a polyesterase which degrades the PBSA, enabling the fungus to use it as a carbon source [53]. Eight homologues of *HsbA* were discovered in the genome of *M. oryzae*, based on occurrence of the *HsbA* Pfam motif (PF12296). Analysis of HT-SuperSAGE data (Table S14) suggests that four of these *HsbA* encoding genes are differentially up-regulated throughout appressorium development and a further two are up-regulated only at later stages (14–16 hours). These data suggest that *HsbA*-like genes are likely to have a specific role during appressorium development. It may be worth speculating that they are involved in attachment of the developing appressorium to the rice surface and might recruit secreted enzymes that degrade constituents of the plant epidermis (such as, for example, cutinases). *MPG1* (MGG_10315) is expressed at high levels throughout appressorium development, but also during mycelial growth. No transcripts were detected for *MHP1* (MGG_01173), consistent with published data showing very low expression during mycelial growth and appressorium development, but high expression during growth *in planta* [52]. The two other hydrophobin encoding genes did not show differential expression during appressorium development.

The HT-SuperSAGE data described in this study have been made easily accessible to the wider research community by submission to Genbank, but also by creation of an online database (http://cogeme.ex.ac.uk/supersage/), as part of the suite of COGEME databases. The user enters the ID of any *M. oryzae* gene and the database will provide HT-SuperSAGE data for the time course of appressorium development, as well as data from a Δ*pmk1* mutant and mycelial growth for comparison. In this way, the expression profiles of more than 96% of the *M. oryzae* genome can be evaluated during infection-related development.

## Discussion

NGS has revolutionised transcriptomic analysis, allowing study of gene expression with a hitherto unattainable level of resolution. RNA-Seq is a powerful tool for visualising transcriptome complexity, enabling genome-wide identification of coding sequences, gene structures, alternative splicing and non-coding RNAs [26]. It can also be used to quantify transcript abundance. Digital gene expression (DGE), in which 21 base tags from 3′-ends of genes are generated directly from cDNA and sequenced using NGS, is more affordable for comparative gene expression studies [54]. A previous comparison between the two techniques estimated that for 90% coverage of the human transcriptome, more than eight times as many RNA-Seq reads would be needed as compared to DGE reads [30]. In fact, the two technologies are complementary and in a transcriptomic study of a bacteria-challenged marine fish (*Lateolabrax japonicus*), RNA-Seq was used first to identify the structure and variation in the transcriptome and DGE then used to quantify expression levels of individual genes [54]. In this study we used a variation of DGE, known as HT-SuperSAGE that generates longer 26 base tags, thus facilitating unambiguous matching of tags to gene sequences [27]. The protocol allows multiple samples to be analysed on a single lane of an Illumina flow-cell, using 4 bp ‘bar-codes’ to identify tags from different samples, thus reducing running costs. We found this an effective means of determining the transcriptional profile of more than 96% of the predicted gene set from *Magnaporthe oryzae* during appressorium formation.

In common with many important plant pathogenic fungi, *M. oryzae* elaborates a specialised infection structure, the appressorium, to enable it to penetrate the host epidermis [19]. The appressorium develops from the end of a germ tube that grows from a three-celled spore, the conidium, which adheres to the surface of a rice leaf. The appressorium generates high turgor, which is used to create mechanical force to penetrate the plant cuticle and enter the underlying epidermal cells. In this study, we used HT-SuperSAGE to analyse global patterns of gene expression during appressorium formation and elucidate physiological pathways important for appressorium development and function. We analysed appressorium differentiation on artificial surfaces so that all gene expression data generated would be exclusively from *M. oryzae* rather than its rice host. Our rationale for doing this was because we wanted to define appressorium-associated gene expression primarily, as a first step in understanding global patterns of gene expression during plant infection by the fungus. It is technically difficult to identify *M. oryzae* gene expression during the early stages of rice infection due to the paucity of fungal material present compared to rice tissue. Our coverage of 96% of the predicted genes means an almost complete coverage of the *M. oryzae* genome that, coupled with the extreme depth provide by the NGS technologies employed, has provided for a level of statistical rigor that cannot be approached by studies using other presently available transcriptomic platforms. Additionally, this has allowed us to identify components of complete metabolic pathways of potential interest. In due course, we will need to analyse these data sets within the wider context of pathogen and host gene expression during infections on living rice plants, but it is clear that the experimental design and methods employed have allowed us to identify the most significant changes in gene expression associated with formation of a functional appressorium by *M. oryzae*.

Germination of conidia and formation of appressoria occurs in the absence of exogenous nutrients and therefore relies on conidial storage compounds for cell growth and the synthesis of compatible solutes, such as glycerol, necessary for development of turgor in the appressorium. During appressorium development, Pmk1-dependent mobilisation of lipids and glycogen has been observed [14]. This is accompanied by an increase in triacylglycerol activity, which liberates glycerol from stored lipids. An addition, both the beta-oxidation pathway and glyoxylate cycle are important for the formation of functional appressoria [15], [16]. Together these two pathways allow fatty acids to be broken down and the carbon units from these compounds can be used to synthesise sugars and glycerol via gluconeogenesis. Acetyl-CoA can be inferred to be an important compound in the metabolic changes that occur during appressorium formation, being the link between the beta-oxidation pathway and the glyoxylate shunt and is also needed for synthesis of melanin (which is necessary for the generation of turgor in the appressorium) and chitins and glucans necessary for cell wall biogenesis. The importance of acetyl-CoA in appressorium morphogenesis has been confirmed by studies showing that mutants of *M. oryzae* lacking carnitine acetyl transferase activity are unable to undergo appressorium-mediated plant infection [17], [18]. Oh, *et al.*, [22] noted the significance of the altered expression of genes related to fatty acid catabolism and the potential importance of the peroxisome in appressorium maturation. By analysing differential expression of genes encoding enzymes that either utilise or produce acetyl-CoA, we have presented evidence here that during appressorium formation acetyl-CoA is synthesised mainly by beta-oxidation of fatty acids. Acetyl-CoA is likely used to synthesise polyketides (particularly melanin) and also fed into gluconeogenesis via the glyoxylate cycle. Carnitine acetyl transferase encoding genes are also differentially expressed during this process, providing further evidence of the importance of acetyl-CoA movement across peroxisomal and mitochondrial membranes during appressorium formation [17], [18]. Consistent with the major role of the Pmk1 MAP-kinase pathway in controlling appressorium morphogenesis, is the observation that genes encoding melanin biosynthetic enzymes, the multi-functional beta-oxidation enzyme *MFP1* and carnitine acetyl transferases were reduced in expression in a Δ*pmk1* mutant compared to Guy11 during early stages of appressorium formation.

This study has also provided evidence that a large set of transporter-encoding genes is differentially expressed during appressorium formation. Sugar transporter genes and secreted oligosaccharide-degrading enzymes are up-regulated, suggesting that *M. oryzae* uses the appressorium to prepare for tissue invasion and use of host plant carbohydrates as a source of nutrition. In particular, we were interested to find clues to the likely major substrates used by *M. oryzae* during plant infection. The observation that quinate permeases are up-regulated as well as genes from the quinate utilisation cluster, strongly suggests that quinate produced in rice cells may be used by *M. oryzae* as a major carbon source. This is consistent with a metabolomic study which identified significant increases in quinate within blast-infected leaf tissue, suggesting pathogen-mediated alteration of host plant metabolism to increase synthesis of quinate during infection [44]. Quinate is converted to protocatechuate by three reactions, catalyzed by quinate dehydrogenase, dehydroquinate dehydratase, and dehydroshikimate dehydratase, respectively. Subsequently, protocatechuate is metabolized through the β-ketoadipate pathway. All of the *M. oryzae* quinate utilization genes are differentially expressed during appressorium development. Critically, dehydroquinate and dehydroshikimate are also intermediates of the shikimate pathway, which leads to branched pathways of biosynthesis of various aromatic amino acids, vitamins, and quinones, as well as plant defense compounds via the phenylproanoid pathway. Diverting the shikimate pathway to produce quinate, for uptake and metabolism by *M. oryzae*, provides a means of potentially suppressing plant defense. Recent evidence has shown that such metabolic priming may play a significant role in effector-mediated suppression of plant defenses [55]. In the corn smut fungus *Ustilago maydis*, for instance, a chorismate mutase is deployed by the fungus to reduce salicylic acid biosynthesis [55]. *M. oryzae* expresses an isochorismatase that might also serve such a purpose (Table S2), as well as possessing a chorismate mutase. However, the systematic, co-ordinated regulation of quinate permeases and quinate metabolic enzymes provides strong evidence for an effective means of suppressing plant defense and fueling fungal growth by the rice blast fungus that will need to be tested by gene functional analysis. Another striking observation was the up-regulation of a wide range of sugar transporters and putative efflux pumps and ABC transporters during appressorium maturation. This fact points to large-scale deployment of fungal secondary metabolites during plant tissue colonization and utilization of a significant family of membrane-bound pumps to contend with corresponding plant defense compounds. The repertoire is likely to be distinct from those used by *M. oryzae* during mycelial growth given the extensive pattern of differential gene expression. The analysis presented in this paper has highlighted a group of hydrophobic surface binding proteins of the same family as *HsbA* from *A. oryzae* [53] that show significant up-regulation of expression during appressorium development, suggesting they may play an important role in this process, potentially by recruiting hydrolytic enzymes to the fungal cell surface. This study has demonstrated the value of NGS sequencing technologies in studying gene expression during a morphogenetic process that is vital for fungal pathogenesis. We have used this data to create a publicly available resource that can be accessed at http://cogeme.ex.ac.uk/supersage/, providing the means for any *M. oryzae* gene to be readily interrogated for its expression profile during infection-related development.

## Materials and Methods

### Fungal strains and growth conditions

*M. oryzae* strains Guy11 [56] and Δ*pmk1* [11]were used in this study. For RNA preparation, mycelia were grown in shaking culture in complete medium, CM [24] for 36 h at 25°C, 200 rpm and harvested by filtering through 3 layers of Miracloth (EMD Biosciences), washed and frozen in liquid nitrogen. For growth in glucose minimal medium (GMM), mycelia growing in CM for 36 hrs were washed, transferred to GMM, grown for an additional 16 hrs and harvested as above. Conidia were harvested from 14-day old CM agar plates and washed three times with sterile water. For germination, conidia were diluted in sterile water to 7.5×10^5^ conidia / ml in the presence of 50 ng/µl 1,16-Hexadecanediol. This solution was then used to flood plastic coverslips (Cole-Parmer) previously glued to square petri plates (Greiner Bio One). Formation of appressoria was monitored under a light microscope and samples were collected at 4, 6, 8, 14 and 16 h by scraping the surface of the coverslips with a sterile razor blade. Recovered samples were immediately frozen in liquid nitrogen, lyophilized and stored at −80°C until needed.

### RNA extraction

Total RNA was extracted from mycelia or germinating conidia using the Qiagen RNeasy Plant Mini kit according to manufacturer's instructions. RNA was eluted in RNase-free water and checked for integrity and quantity on an Agilent 2100 Bioanalyzer according to manufacturer's instructions. RNA with integrity number of at least 6.5 was used for library preparations. RNA was prepared from at least two biological replicates and used for independent library preparations.

### Library preparation for RNA-Seq

Sequencing libraries were prepared using mRNA-Seq Sample Preparation kit from Illumina from 9 µg of total RNA according to the manufacturer's instructions. Libraries were quantified and checked for quality on Agilent 2100 Bioanalyzer using a DNA 1000 chip kit. Each library was diluted to 10 nM in Elution Buffer (Qiagen) and used for sequencing using an Illumina Genome Analyser GX II platform.

### Library preparation for HT-SuperSAGE

Individual sequencing libraries were prepared for germinating conidia at each individual time point as well as mycelia grown in CM and GMM (two biological replicates for each sample). Additional libraries were also prepared for germinating conidia from the Δ*pmk1* mutant harvested at 4 hr after plating. These libraries were prepared from 10 µg total RNA according to the method described previously [27] with minor modifications. Libraries of tagged cDNA fragments were PCR amplified using Hot Start Phusion DNA polymerase and GEX-1 and -2 primers (Table S13) for 15 cycles according to the following parameters; initial denaturation at 98°C for 1 min followed by 15 cycles of 98°C for 10 sec; 62.5°C for 20 sec; 72°C for 30 sec and a final extension at 72°C for 2 min. PCR products were ethanol precipitated, re-suspended in 25 µl LoTE and size separated on 8% non-denaturing polyacrylamide gels using TAE buffer. Products were visualised by ethidium bromide staining and 123–125 bp sized products were excised from the gel. Products were then extracted from gel in EB, quantified on Agilent Bioanalyzer 2100 using DNA 1000 chip kit and adjusted to 10 nM final concentration in EB. Products were ligated to indexed Adapters-1 (Table S13). These adapters contain defined index sequences for sample identification (Table S14), enabling four samples to be analysed per lane of an Illumina flow cell. Four libraries thus prepared were pooled and used for sequencing as with the RNA-Seq libraries.

### Analysis of RNA-Seq

Tophat software [57] was used to align short reads to the published genome of *Magnaporthe oryzae*, version 6 (http://www.broadinstitute.org/annotation/genome/magnaporthe_grisea/MultiHome.html) [3] and to predict exon splice sites. Cufflinks software [58] was used to analyse this data (using reads with a mapping quality >30) from both biological replicates along with gene annotations from *M. oryzae*, resulting in normalised counts (expressed in fragments per kilobase of exon model per million mapped fragments – FPKM) for each gene. The Cuffdiff component of the Cufflinks package was used to look for significant differences in FPKM between different samples.

### Analysis of tag frequency for HT-SuperSAGE

FASTX Barcode Splitter from the FASTX-Toolkit (http://hannonlab.cshl.edu/fastx_toolkit/download.html) was used to separate the samples from the same lane using the four base barcode. FASTA/Q trimmer from the FASTX-Toolkit was used to remove the 4 base barcode from the sequence and then to remove the sequence from position 27 to the end of the sequence leaving a 26 base tag sequence. Any remaining adapter sequences were removed using FASTA/Q clipper from the FASTX-Toolkit. FASTQ-to-FASTA from the FASTX-toolkit was used to convert the tag sequences to a FASTA format. The frequency of each tag was calculated using custom perl scripts. Tags were mapped to predicted transcripts from the published genome of *Magnaporthe oryzae* (version 6) using Bowtie [59], allowing one base mismatch. For each transcript, the frequencies of all the tags mapped to that gene were summed. Statistical analysis or data was performed using DESeq [60]. Transcript abundances for each gene were expressed as a weighted mean of counts from each replicate normalised to overall library size (known as ‘base mean’). P-values (adjusted for false discovery rate) were generated for each gene in pair-wise comparisons between different condtions. In our analyses, we used an adjusted P-value of < = 0.05 as a criteria for identifying significant differences in gene expression.

### Database resource

HT-SuperSAGE data (raw counts and TPM) obtained from Guy11 mycelium grown in CM, time course of appressorium development in Guy11 and Δ*pmk1* mutant conidia left to germinate for 4 hours was stored in a MySQL database. A publicly available web-based front end was constructed for this database which can be accessed at http://cogeme.ex.ac.uk/supersage/.

### Data availability

HT-SuperSAGE and RNA-Seq data described in this paper has been submitted to Gene Expression Omnibus (GEO) at NCBI (http://www.ncbi.nlm.nih.gov/geo/), accession numbers GSE30069, GSE30256, GSE30327.

## Supporting Information

Figure S1 **Heatmap showing levels of transcript abundance from genes encoding autophagy-related proteins during time course of appressorium development in** ***M. oryzae*** **.** Levels of expression are represented as moderated log_2_ ratio of transcript abundance compared to *M. oryzae* mycelium grown in complete medium. Values are from 4 h (T4) to 16 h (T16) after conidia are placed on a hydrophobic surface. Genes showing similar patterns of gene expression have been clustered.
(TIF)

Table S1 **HT-SuperSAGE data showing** ***M. oryzae*** **genes differentially expressed during early appressorium development (4–8 h) vs mycelial growth in CM (adjusted P-value< = 0.05).**
(XLS)

Table S2 **HT-SuperSAGE data showing** ***M. oryzae*** **genes differentially expressed during appressorium maturation (14–16 h) vs mycelial growth in CM (adjusted P-value< = 0.05).**
(XLS)

Table S3 **HT-SuperSAGE data showing** ***M. oryzae*** **genes differentially regulated in a** ***Δpmk1*** **mutant (P4) compared to Guy11 (T4) during early appressorium development (4 h) (adjusted P-value< = 0.05).**
(XLS)

Table S4 **HT-SuperSAGE data showing** ***M. oryzae*** **genes differentially regulated in mycelium grown in minimal medium (MM) compared to mycelium propagated in complete medium (CM) (adjusted P-value< = 0.05).**
(XLS)

Table S5 **HT-SuperSAGE data showing differential expression of** ***M. oryzae*** **genes encoding enzymes that directly utilise or produce acetyl-CoA during appressorium morphogenesis.**
(XLS)

Table S6 **HT-SuperSAGE data showing differential expression of** ***M. oryzae*** **genes encoding enzymes that utilise malonyl-CoA during appressorium morphogenesis.**
(XLS)

Table S7 **HT-SuperSAGE data showing differential expression of** ***M. oryzae*** **genes encoding enzymes involved in fatty acid β-oxidation and the glyoxylate cycle during appressorium morphogenesis.**
(XLS)

Table S8 **HT-SuperSAGE data showing differential expression of** ***M. oryzae*** **genes encoding secreted glycosyl hydrolases, carbohydrate esterases and polysaccharide lyases during appressorium morphogenesis.**
(XLS)

Table S9 **HT-SuperSAGE data showing differential expression of** ***M. oryzae*** **genes encoding sugar transporters during appressorium morphogenesis.**
(XLS)

Table S10 **HT-SuperSAGE data showing differential expression of** ***M. oryzae*** **genes encoding enzymes involved quinate utilisation and shikimate pathway during appressorium morphogenesis.**
(XLS)

Table S11 **HT-SuperSAGE data showing differential expression of** ***M. oryzae*** **genes encoding organic acid transporters during appressorium morphogenesis.**
(XLS)

Table S12 **HT-SuperSAGE data showing differential expression of** ***M. oryzae*** **genes encoding drug transporters during appressorium morphogenesis.**
(XLS)

Table S13 **HT-SuperSAGE data showing differential expression of** ***M. oryzae*** **genes encoding key secondary metabolic enzymes during appressorium morphogenesis.**
(XLS)

Table S14 **HT-SuperSAGE data showing differential expression of** ***M. oryzae*** **genes encoding hydrophobins and HsbA like-proteins during appressorium morphogenesis.**
(XLS)
