# Supplementary figures and images for: Genome-wide Transcriptional Profiling of Appressorium Development by the Rice Blast Fungus Magnaporthe oryzae
Source: PLoS Pathog. 2012 Feb 9;8(2):e1002514. doi: 10.1371/journal.ppat.1002514 (PMC3276559; doi:10.1371/journal.ppat.1002514)

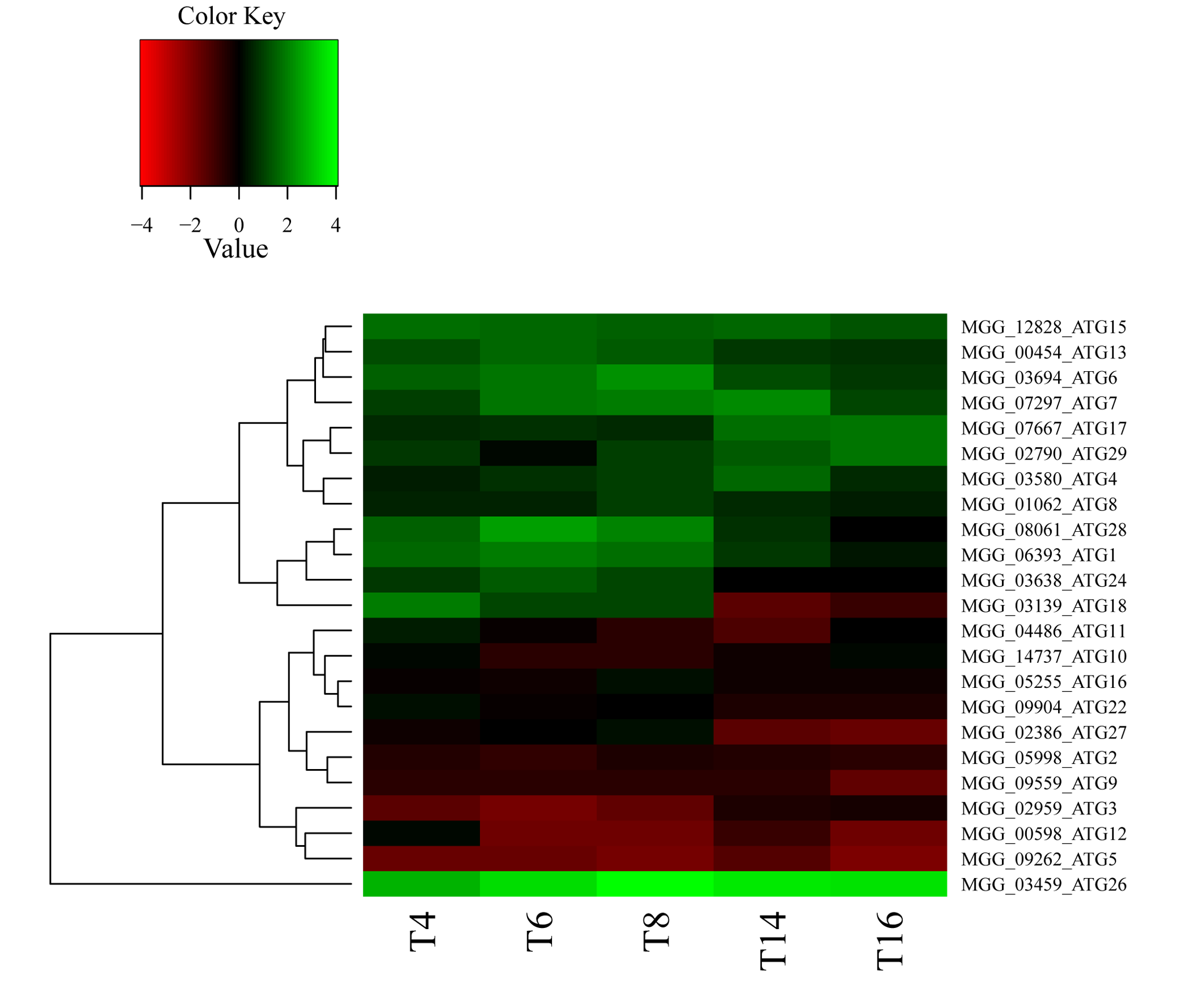

Supplement: Figure S1 — Heatmap showing levels of transcript abundance from genes encoding autophagy-related proteins during time course of appressorium development in M. oryzae . Levels of expression are represented as moderated log2 ratio of transcript abundance compared to M. oryzae mycelium grown in complete medium. Values are from 4 h (T4) to 16 h (T16) after conidia are placed on a hydrophobic surface. Genes showing similar patterns of gene expression have been clustered. (TIF) [file ppat.1002514.s001.tif]
